# The clinical value of local consolidative therapy for oligo-residual disease in PD-1/PD-L1 inhibitors-treated non-small cell lung cancer

**DOI:** 10.3389/fimmu.2024.1525236

**Published:** 2024-12-17

**Authors:** Yuqi Su, Pan Luo, Ling Ni, Jianbin Hu, Jie Weng, Erdong Shen, Qiang Zhou, Tao Chen, Jiwen Xiao, Jia Xiao, Wangti Xie, Rong Shan, Xiang Yao, Fang Wen

**Affiliations:** ^1^ Department of Oncology, Yueyang Central Hospital, Yueyang, Hunan, China; ^2^ Department of Thoracic Surgery, Yueyang Central Hospital, Yueyang, Hunan, China; ^3^ Department of Oncology, Hunan University of Medicine General Hosipital, Huaihua, Hunan, China

**Keywords:** non-small cell lung cancer, local consolidative therapy, PD-1/PD-L1 inhibitors, oligo-residual disease, immune checkpoint inhibitors

## Abstract

**Background:**

Few real-world studies exist regarding the clinical value of local consolidative therapy (LCT) for oligo-residual disease (ORD) in NSCLC patients treated with immune checkpoint inhibitors. Therefore, we retrospectively evaluated whether LCT could improve the prognosis of NSCL patients with ORD after effective first-line PD-1/PD-L1 inhibitors treatment.

**Methods:**

A total of 132 patients with metastatic NSCLC who had received first-line PD-1/PD-L1inhibitors-based systemic treatment and developed ORD (defined as residual tumors limited to three organs and five lesions) were included. The LCT group consisted of 41 patients received LCTs for oligo-residual lesions before treatment failure, and the remaining 91 patients, who did not receive local therapies, constituted the non-LCT group. The progression-free survival (PFS) and overall survival (OS) of the two groups were analyzed.

**Results:**

With a median follow-up of 12.0 months, 86 patients developed progressive disease and 42 patients died. Compared with the non-LCT group, LCT group exhibited significant longer progression-free survival (PFS) (median 11.0 vs. 7.0 months, P=0.017) and overall survival (OS) (median 26.0 vs. 17.0 months, P=0.003). Multivariable analysis demonstrated that LCT was an independent predictor of prolonged PFS (HR=0.606, 95% CI=0.370–0.964, P=0.035) and OS (HR=0.467, 95% CI=0.229–0.949, P=0.035). Subgroup analysis revealed that the dominant population considerably benefited from LCT in terms of PFS and OS included patients with 1-2 residual tumor sites (mPFS: 11.0 vs. 7.0 months, P=0.013; mOS: 23.0 vs. 17.0 months, P=0.018) and those with high PD-L1 expression (mPFS: 13.0 vs. 7.0 months, P=0.018; mOS: 34.0 vs. 16.0 months, P=0.030). In addition, the All-LCT group had significantly longer PFS (mPFS 16.0 vs. 7.0, P=0.002) and OS (mOS 28.0 vs. 17.0, P= 0.002) than did the non-LCT group. However, patients who received LCT to only some of their lesions had not experienced improvements in PFS (P=0.546) or OS (P=0.198).

**Conclusion:**

LCT may provide extra survival benefits among patients with oligo-residual NSCLC after effective first-line PD-1/PD-L1 inhibitors treatment, particularly in those patients with one or two residual lesions, high PD-L1 expression, or who had received LCT for all lesions. LCT may be a novel treatment option for this specific population.

## 1 Introduction

Lung cancer is the most common malignancy, and its annual morbidity and mortality rates continue to increase. Non-small cell lung cancer (NSCLC) accounts for more than 80% of lung cancers, and approximately 50% of NSCLC patients present with distant metastases at diagnosis (1, 2). Advanced NSCLC patients who lack active anaplastic lymphoma kinase (ALK) or epidermal growth factor receptor (EGFR) genetic mutations are considered to have a poor prognosis. However, PD-1/PD-L1 inhibitors, which target the programmed death ligand-1 (PD-L1) or programmed death-1 (PD-1) axis, have recently dramatically changed the treatment scenario for these patients (3–5). Several pivotal trials have demonstrated that PD-1/PD-L1 inhibitors (involving either monotherapy or combination therapy) improve the clinical efficacy of first-line treatment for advanced NSCLC patients without ALK or EGFR mutations (6–8), especially for those with PD-L1 expression ≥ 50% (9, 10). However, the development of resistance to monotherapy remains the main obstacle to achieving long-term survival in advanced NSCLC patients.

Local consolidative therapy (LCT) has been defined as any palliative or radical local therapy directed at the primary site of disease or any site of metastasis. LCTs for lung cancer include surgical resection, definitive radiotherapy and ablation. The clinical role of LCT in advanced NSCLC has been extensively studied. Many studies have shown that LCT can improve treatment outcomes for select patients with advanced lung cancer (11–13). In 2017, an analysis of the Surveillance, Epidemiology, and End Results (SEER) database suggested that multimodality therapy, particularly involving combined thoracic surgery and chemotherapy, can dramatically improve the prognosis of patients with stage IV NSCLC (14). A multicenter randomized trial led by the MD Anderson Cancer Center showed that LCT improved PFS and OS compared with systemic maintenance treatment (15). Thoracic or hepatic ablation, including microwave ablation (MWA) and radiofrequency ablation, has been shown to improve the prognosis of metastatic NSCLC (16–18) and is thought to be as effective as surgery in early-stage NSCLC (19–21). Surgical interventions at the primary or metastatic site of NSCLC provide remarkable benefits when combined with standard systemic treatment for advanced NSCLC patients (22–24). SBRT (stereotactic body radiation therapy) relieves clinical symptoms and effectively controls locally progressing lesions in advanced NSCLC patients (25). However, whether SBRT improves the prognosis of advanced NSCLC has been controversial. Some studies have demonstrated no significant improvement in the treatment response or prognosis resulting from the addition of SBRT to PD-1/PD-L1 inhibitors in advanced NSCLC patients compared with the use of PD-1/PD-L1 inhibitors alone (26, 27).

The concept of the oligo-residual disease (ORD) state is inspired by the oligo-metastatic paradigm, which is different from widely used methods for treating metastatic disease. Oligo-residual sites may be the main progressive lesions when patients are resistant to systematic treatment (28). Limited research has shown that LCT can improve local control and increase PFS when it is administered for oligo-residual disease in advanced NSCLC patients receiving TKIs (29). However, the outcome of the real-world application of LCT in immune checkpoint inhibitors-treated NSCLC patients with oligo-residual disease has been rarely reported. Herein, we conducted a retrospective cohort study to investigate whether LCT combined with PD-1/PD-L1 inhibitor is a promising first-line strategy for oligo-residual advanced NSCLC.

## 2 Materials and methods

### 2.1 Patients

Metastatic NSCLC patients without EGFR or ALK genetic mutations who received first-line PD-1/PD-L1 inhibitors monotherapy or its combination treatment at the Yueyang Center Hospital and Hunan University of Medicine General Hosipital between March 2019 and February 2024 were retrospectively reviewed. ORD was defined as the presence of 1-5 residual lesions (including the primary site, cranial or lymph node metastases were counted per lesion) limited to 1-3 organs at the best response of PD-1/PD-L1 inhibitors treatment, according to the consensus of oligometastatic disease (30). Lesions that had completely disappeared on computed tomography (CT) or magnetic resonance imaging (MRI) were excluded from the definition of residual disease. The inclusion criteria were as follows: 1) patients with a pathological diagnosis; 2) patients who received first-line PD-1/PD-L1 inhibitors monotherapy or its combination with other therapies; 3) patients with an oligo-residual disease status that occurred during the PD-1/PD-L1 inhibitors treatment period; 4) patients with tumors that were evaluated via brain magnetic resonance imaging (MRI) and chest and abdomen enhanced CT scan or 18F-fluorodeoxyglucose positron emission tomography (18F-FDG-PET-CT) at the time of definitive ORD determination or initial staging; and 5) patients who were ≥18-years-old and who had an Eastern Cooperative Oncology Group (ECOG) performance status (PS) score<2. The exclusion criteria were as follows: 1) patients with a history of other malignancies; 2) patients with oligo-metastatic disease at baseline; 3) patients with pleural metastasis or pericardial effusion; 4) patients who received LCT within three weeks of or before initiating PD-1/PD-L1 inhibitors therapy; and 5) patients with PFS ≤2 months. The study was conducted in accordance with the Declaration of Helsinki and was ethically approved by the institutional review boards of the two medical centers (approval number: 2022-049).

### 2.2 Treatment and follow-up

The decision to use PD-1/PD-L1 inhibitors as monotherapy or in combination with other therapies was based on physician recommendations and patient preferences. PD-1/PD-L1 inhibitors were administered every 3 weeks until the onset of intolerable toxicity or disease progression (up to 24 months) and mainly included tislelizumab (200 mg/cycle), camrelizumab (200 mg/cycle), sintilimab (200 mg/cycle), pembrolizumab (200 mg/cycle) and atezolizumab (1,200 mg/cycle). LCT, including hypofractionated or conventional radiotherapy, surgery and other local ablative therapies, was performed on some or all of the oligo-residual primary or metastatic sites after effective systemic treatment and before initial disease progression, according to the advice of the multidisciplinary team and with patient consent. Chest and abdominal enhanced CT scans were performed every 6 weeks for therapeutic response evaluation. Enhanced brain magnetic resonance imaging (MRI) was performed every 6 weeks if there was evidence of brain metastases and approximately every 4 months if there was no baseline brain lesion and no symptoms thereafter. Serial images of the patients were reviewed by a senior radiologist. Telephone calls were also used to obtain medical records, and the cut-off date was 10 July 2024.

### 2.3 Outcomes and statistical analysis

Overall survival (OS) was defined as the time interval from diagnosis to the date of last follow-up or death. Progression-free survival (PFS) was defined as the time from treatment initiation to treatment and disease progression (according to Response Evaluation Criteria in Solid Tumours [RECIST], version 1.1) or censoring if there was no disease progression or death; moreover, patients without disease progression at the end of the study were recorded as censored.

Statistical analyses were performed via SPSS 23.0 software. PFS and OS were estimated via the Kaplan-Meier method, and survival curves were compared via log-rank tests. GraphPad Prism 8.0 was used to plot survival curves. Cox proportional hazards regression analyses were used to evaluate prognostic factors affecting PFS and OS and to calculate hazard ratios (HRs) for OS and PFS. P value less than 0.05 (two-sided) was considered to be statistically significant in this study.

## 3 Results

### 3.1 Patient characteristics

A total of 132 patients, consisting of 71 males and 61 females, were included in our study (**Table 1**). Ninety-one patients received only systemic therapy (non-LCT group), whereas the other 41 patients received local consolidative therapy at the best response after effective systemic treatment (LCT group, detailed in **Table 2**). Local ablative therapy was administered to the small-sized lung (18 pts) and liver (6 pts) lesions. Hypofractionated radiotherapy (>2 Gy to <5Gy per fraction) was more frequently used for the treatment of bone (5 pts) and intracranial lesions (3 pts), whereas stereotactic body radiotherapy (SBRT, ≥5 Gy per fraction) was mainly used for patients with small intracranial (8 pts) or lung lesions (9 pts). Surgical therapy was mainly performed for solitary metastatic lesion (3 pts) or primary lung lesion (2 pts). LCT was performed at all sites of residual disease (complete LCT, including the primary tumor and all metastases) in 36.6% (15/41) of the patients in our study cohort. Among these 132 patients, 51 (38.6%) had high PD-L1 expression (≥50%), whereas 33 (25.0%) had low PD-L1 expression (<50%). For the remaining 48 (36.4%) patients, data on PD-L1 expression status were not available, or PD-L1 testing was not performed.

**Table 1:** Patients baseline characteristics.

values	No.(%)	LCT group	non-LCT group	*P* value^*^
Age (years)				0.258
≤55	74(56.06%)	20	54	
>55	58(43.94%)	21	37	
Gender				0.810
Male	71(53.79%)	14	57	
Female	61(46.21%)	27	34	
Smoking				0.304
Yes	57(43.18%)	15	42	
No	75(56.82%)	26	49	
Pathology				0.319
Squamous cell carcinoma	60(45.45%)	16	44	
Non-squamous carcinoma	72(54.55%)	25	47	
Regimens				0.818
Combinational therapy	56(42.42%)	18	38	
Monotherapy	76(57.58%)	23	53	
ICIs^†^				0.869
PD-L1 monoclonal antibody	31(23.48%)	10	21	
PD-1 monoclonal antibody	101(76.52%)	31	70	
Number of residual sites				0.442
1-2	72(54.55%)	24	48	
3-5	60(45.45%)	16	43	
Number of metastatic organs				0.741
1-2	68(51.52%)	22	46	
>2	64(48.48%)	19	45	
Brain metastasis				0.993
Yes	87(65.91%)	27	60	
No	45(34.09%)	14	31	
PD-L1 TPS (%)				0.814
Low+unknown	84(63.64%)	28	56	
≥50%	48(36.36%)	16	35	

^*^Chi-square test; **^†^** Immune checkpoint inhibitors.

**Table 2:** Detailed information of local consolidative therapy of patients with ORD.

	All (n = 41) n%*
Radiotherapy	
Conventionally fractionated external-beam radiation	
Spinal cord metastasis	1(2.4%)
Adrenal gland metastasis	2(4.9%)
Lymphatic metastasis	3(7.3%)
Primary or metastatic lung lesion	2(4.9%)
Stereotactic body radiotherapy	
Intracranial metastasis	8(19.5%)
Bone metastasis	3(7.3%)
Primary or metastatic lung lesion	9(22.0%)
Hypofractionated radiotherapy	
Intracranial metastasis	3(7.3%)
Bone metastasis	5(12.2%)
Primary or metastatic lung lesion	1(2.4%)
Surgical resection	
Intracranial metastasis1(2.4%)	
Lymphatic metastasis	1(2.4%)
Primary or metastatic lung lesion	2(4.9%)
Adrenal gland metastasis	1(2.4%)
Ablation	
Microwave ablation	
Primary or metastatic lung lesion	6(14.6%)
Liver metastasis	2(4.9%)
Radiofrequency ablation	
Primary or metastatic lung lesion	3(7.3%)
Liver metastasis	4(9.8%)
Cryoablation	
Primary or metastatic lung lesion	9(22.0%)

ORD, oligo-residual disease.

### 3.2 Survival outcomes of the overall population

After a median follow-up time of 12.0 months (95% confidence interval [CI]=9.98–14.02), 46 patients did not progress, whereas 42 deaths were recorded (31.82%). Treatment-related adverse events (AEs) in the two groups were not analyzed because of incomplete documentation. However, no grade ≥ 3 local treatment-related adverse events were documented in this study. The median progression-free survival (mPFS) and median overall survival (mOS) times for the entire cohort were 8.0 months (95% CI=6.72–9.28) and 20.0 months (95% CI=16.97–23.03), respectively. Prognostic indicators affecting PFS and OS were identified via univariate (**Supplementary Tables 1**, **2**) and multivariate (**Tables 3**, **4**) Cox regression analyses. According to the results of the multivariate analysis, LCT was an independent factor associated with longer PFS (HR=0.606, 95% CI=0.370–0.964, P=0.035) and OS (HR=0.467, 95% CI=0.229–0.949, P=0.035). LCT led to a 39.4% reduction in the risk of disease progression or death and a 53.3% reduction in the risk of death for oligo-residual NSCLC patients following effective first-line PD-1/PD-L1 inhibitors treatment.

**Table 3:** Multivariate analysis for PFS.

	HR	95% CI	*P* value
Brain metastasis (yes vs. no)	1.582	0.969-2.584	0.067
Number of metastatic organs (1-2 vs. >2)	1.390	0.898-2.153	0.140
Local consolidative therapy (yes vs. no)	0.606	0.370-0.964	0.035

**Table 4:** Multivariate analysis for OS.

	HR	95% CI	*P* value
Age (≤55 vs. >55)	0.447	0.220-0.910	0.026
Number of metastatic organs (1-2 vs. >2)	1.884	0.958-3.705	0.067
Number of residual sites (1-2 vs.3 -5)	1.380	0.713-2.672	0.339
Local consolidative therapy (yes vs. no)	0.467	0.229-0.949	0.035

### 3.3 Prognosis differences between the LCT and non-LCT groups

**Figure 1:**
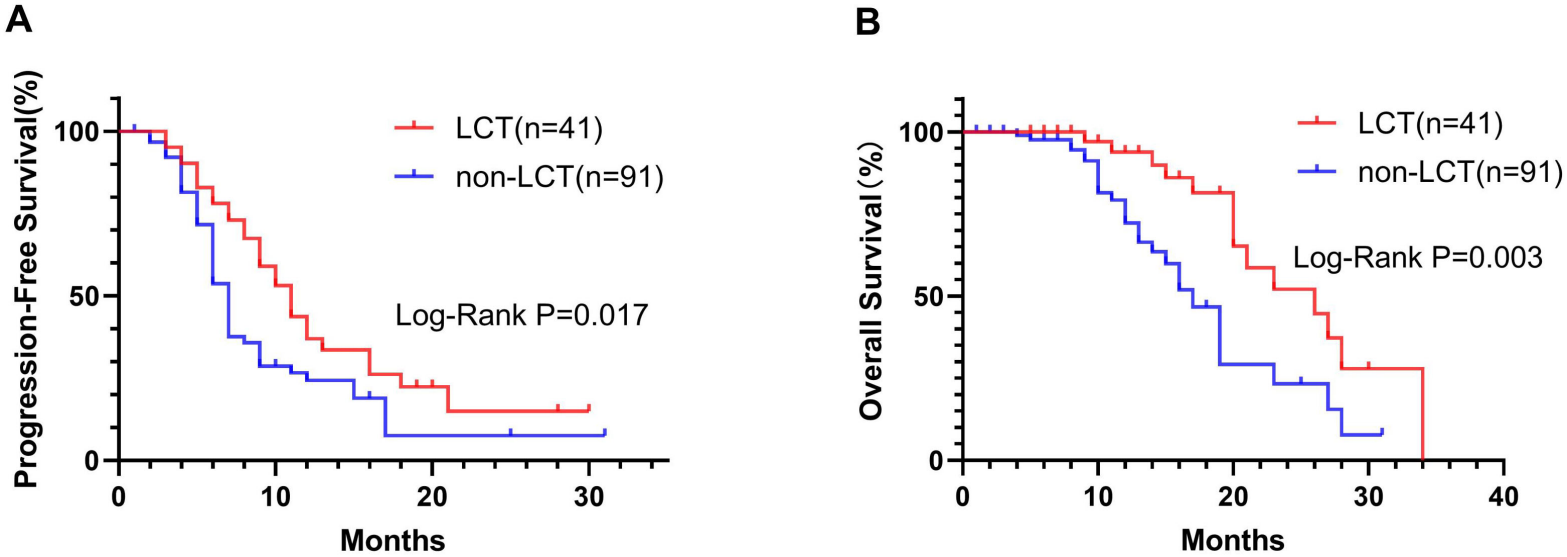
In the Kaplan−Meier plots, the LCT group exhibited longer median progression-free survival (mPFS, **A**) and median overall survival (mOS, **B**) than did the non-LCT group. LCT, local consolidative therapy.

At the last follow-up, 14 (36.15%) and 28 (30.77%) patients had died in the LCT and non-LCT groups, respectively. The Kaplan−Meier curves for the two groups are shown in **Figure 1**. The mPFS in the LCT group (11.0 months, 95% CI 8.86–13.14) was longer than that in the non-LCT group (7.0 months, 95% CI 6.31–7.69) (P=0.017) (**Figure 1A**). Similarly, the mOS was 26.0 months (95% CI 17.46–34.54) in the LCT group, which was longer than the 17.0 months (95% CI 14.68–19.32) in the non-LCT group (P=0.003) (**Figure 1B**). Compared with the non-LCT group, the LCT group clearly exhibited a longer time to disease progression and a better prognosis.

**Figure 2:**
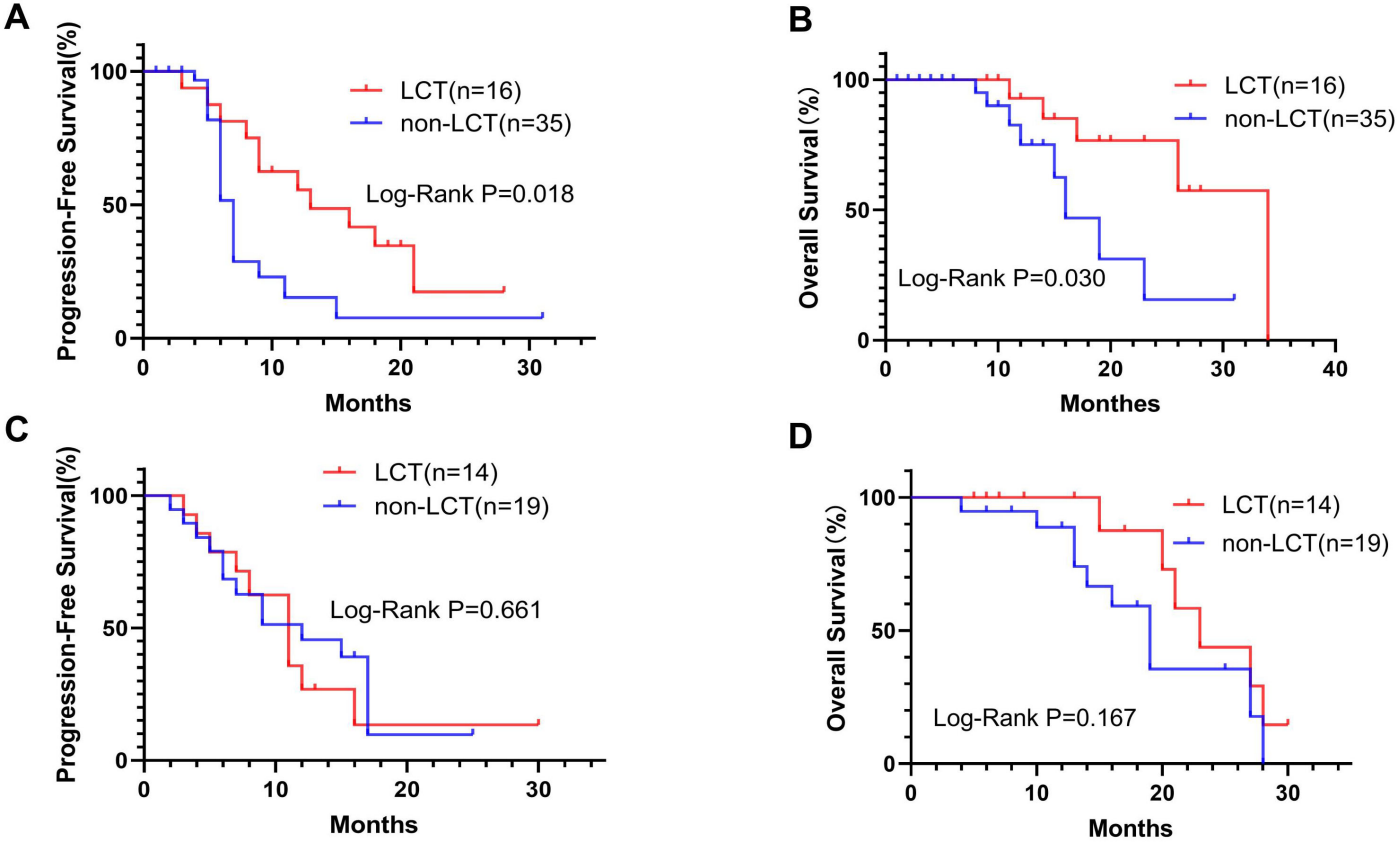
K−M plots showing that LCT prolonged the mPFS **(A)** and mOS **(B)** of patients with high PD-L1 expression, whereas patients with low PD-L1 expression did not benefit from LCT in terms of PFS **(C)** or OS **(D)**. mOS, median overall survival; mPFS, median progression-free survival.

**Figure 3:**
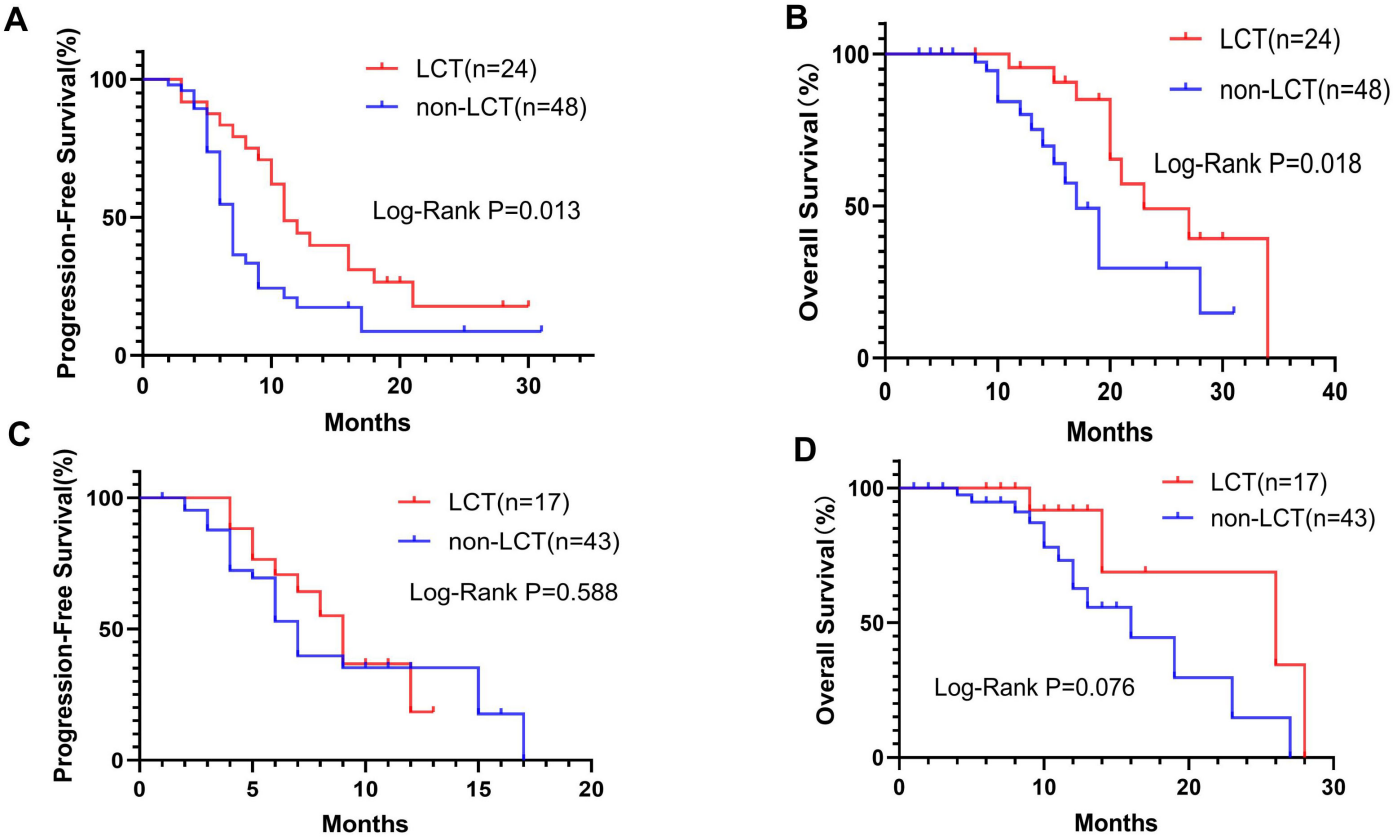
K−M plots. LCT prolonged the mPFS **(A)** and mOS **(B)** for patients with 1–2 residual tumor sites, whereas patients with 3–5 residual tumor sites did not benefit from LCT in terms of PFS **(C)** or OS **(D)**. mOS, median overall survival; mPFS, median progression-free survival.

Subgroup analysis was performed for patients with different PD-L1 levels (**Figure 2**) or numbers of residual lesions (**Figure 3**). LCT significantly prolonged PFS (mPFS: 13.0 vs. 7.0 months, 95% CI 5.78–20.22 vs. 6.24–7.76; P=0.018) and OS (mOS: 34.0 vs. 16.0 months, 95% CI 26.0–NA vs. 11.62–20.38; P=0.030) in patients with high PD-L1 expression (**Figures 2A, B**), whereas patients with low (PFS: P=0.661, OS: P=0.167) (**Figures 2C, D**) PD-L1 expression did not receive any prognostic benefit from LCT. Furthermore, only patients with 1–2 residual lesions benefited from LCT for PFS (mPFS: 11.0 vs. 7.0 months, 95% CI 8.70–13.30 vs. 6.17–7.83; P=0.013) (**Figure 3A**) and OS (mOS: 23.0 vs. 17.0 months, 95% CI 14.29–31.71 vs. 14.44–19.56; P=0.018) (**Figure 3B**). However, LCT did not improve the prognosis in the subgroup with 3–5 oligo-residual lesions (PFS: P=0.588; OS: P=0.076) (**Figures 3C, D**).

### 3.4 Differences in prognosis between the all-LCT and part-LCT groups

**Figure 4:**
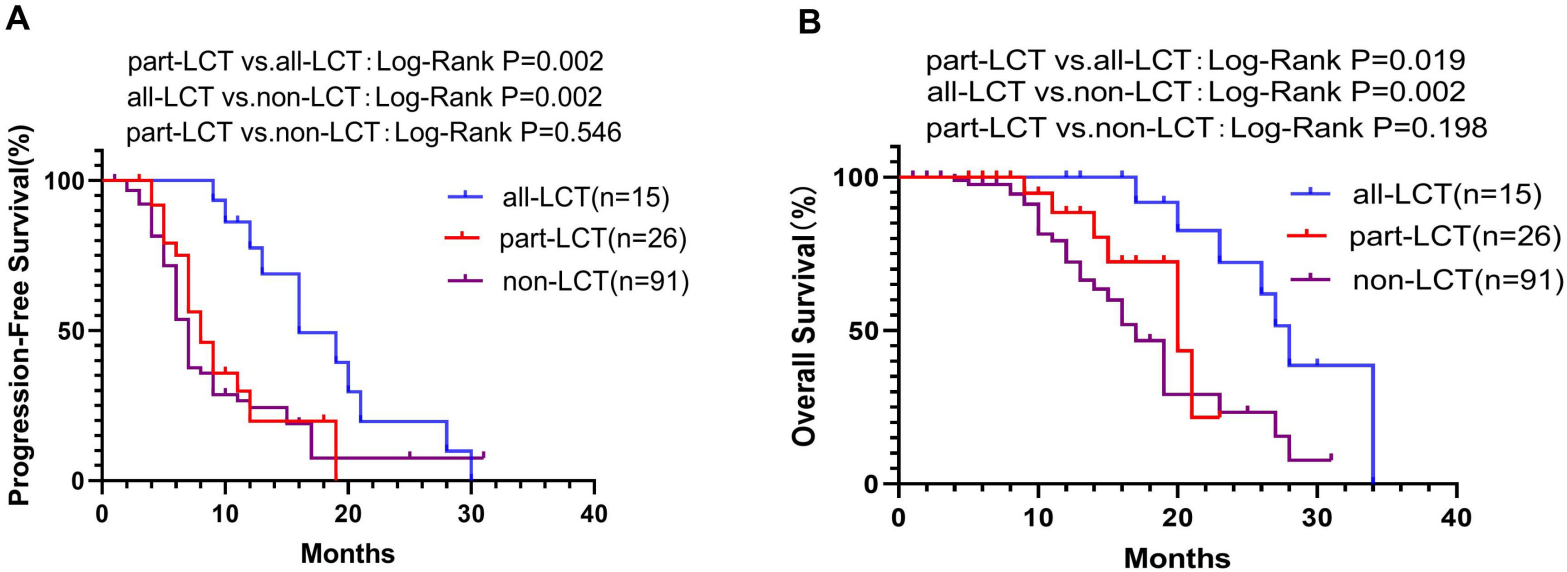
K−M plot of PFS **(A)** and OS **(B)** in the all-LCT, part-LCT and non-LCT groups. OS, overall survival; PFS, progression-free survival.

In the LCT group, 15 patients received complete local consolidative therapy to all of the residual sites (all-LCT group), including both the primary tumor and all of the metastatic lesions, whereas the other 26 patients received LCT to only part of the lesions (part-LCT). PFS in the all-LCT group (mPFS 16.0 months, 95% CI 11.13–20.88) was significantly longer than that in the part-LCT group (mPFS 8.0 months, 95% CI 6.04–9.96; P=0.002) (**Figure 4A**). Patients in the all-LCT group (mOS: 28.0 months, 95% CI 25.25–30.75) exhibited a longer OS time than those in the part-LCT group (mOS: 20.0 months, 95% CI 14.10–25.90; P=0.019) (**Figure 4B**).

In addition, the all-LCT group had significantly longer PFS (mPFS 16.0 vs. 7.0 months, 95% CI 11.13–20.88 vs. 6.31–7.69; P=0.002) (**Figure 4A**) and OS (mOS 28.0 vs. 17.0 months, 95% CI 25.25–30.75; P=0.002) (**Figure 4B**) than did the non-LCT group. However, patients who received LCT to only some of the lesions did not exhibit improved PFS (P=0.546) (**Figure 4A**) or OS (P=0.198) (**Figure 4B**).

## 4 Discussion

LCT, which plays a critical role in preventing local symptoms and complications associated with tumor growth, is widely used in patients with metastatic NSCLC after effective systemic therapy. The role of consolidative radiotherapy in immune checkpoint inhibitors-treated NSCLC patients with ORD demands thorough investigation. To the best of our knowledge, this retrospective study is the first to investigate the real-world use and outcome of LCT for oligo-residual NSCLC patients at the best response of first-line PD-1/PD-L1 inhibitors treatment. We are the first to found that survival benefit of LCT appears to occur mainly in patients with fewer residual lesions, high PD-L1 expression, or receive LCT for all residual diseases. This provides a novel treatment option that should be considered for this subset of patients.

Currently, the survival benefit of local therapy combined with targeted or chemotherapy in patients with oligometastatic NSCLC has been confirmed by many studies, including randomized clinical trials and retrospective studies (15, 23, 25). Previous treatment with radiotherapy in patients with advanced NSCLC results in longer PFS and OS with pembrolizumab treatment than in patients who have not received prior radiotherapy (31). Pembrolizumab after LAT for oligometastatic NSCLC appears to prolong PFS from 7.1 months (as reported in the KEYNOTE-042) to 19.1 months (32, 33). In addition, the PACIFIC trial partially confirmed the survival benefit of immunotherapy combined with radiotherapy in patients with stage III NSCLC. Recently, a randomized phase 2 trial examining the use of SABR alone compared with SABR with immunotherapy (I-SABR) for people with early-stage NSCLC demonstrated that LCT combined with immunotherapy is more favorable for improving the prognosis of early-stage NCSCLC patients (34). Local therapy for oligo-residual lesions has been widely used to relieve clinical symptoms in metastatic NSCLC. However, clinical data on the prognostic value of LCT in patients with PD-1/PD-L1 inhibitors-treated oligo-residual NSCLC are currently scarce and should be further explored in the era of immunotherapy. Moreover, there is a growing need for an appropriate combination strategy to overcome acquired resistance in NSCLC patients receiving PD-1/PD-L1 inhibitor treatment.

Oligo-residual disease is observed in approximately 20% of patients receiving PD-1/PD-L1 inhibitor or targeted therapy (29, 35). Approximately half of patients experience initial progressive disease (PD) exclusively from residual tumor lesions after receiving targeted or PD-1/PD-L1 inhibitor therapies for NSCLC (35, 36). Substantial genetic mutations and immune microenvironmental heterogeneities exist among metastatic NSCLC lesions (37), which may lead to different drug sensitivities among tumor lesions. These drug-resistant lesions may become residual disease and sources of PD. Additionally, some lesions may acquire resistance to PD-1/PD-L1 inhibitors and develop to disease progression ultimately. Local consolidative therapy targeting residual lesions after effective systemic treatment before disease progression is very important for preventing disease progression. Previous studies have demonstrated that LCT can improve local control and significantly prolong PFS in osimertinib-treated NSCLC patients with oligo-residual lesions (29). Our study confirmed the findings in a recent study that LCT appears to offer additional survival benefits for patients with metastatic NSCLC who develop oligo-residual disease following effective immunotherapy (35). Furthermore, we are the first to verify the clinic value of LCT in patients with ORD during first-line PD-1/PD-L1 inhibitor treatment. Compared with systemic treatment alone, LCT combined with PD-1/PD-L1 inhibitor-based first-line treatment led to significant improvements in median PFS (from 7 to 11 months) and median OS (from 17 to 26 months), corresponding to a 39.4% reduction in the risk of recurrence and a 53.3% reduction in the risk of death. Our data (combined with those of previous studies) proved that LCT of residual lesions may be an appropriate combination strategy for overcoming acquired resistance in immunotherapy-treated NSCLC patients.

To better understand the effect of LCT on survival, subgroup analyses based on the number of residual sites or PD-L1 level were performed. Our study is the first to demonstrate that LCT following effective first-line PD-1/PD-L1 inhibitors treatment improved PFS and OS mainly in NSCLC patients with 1–2 residual sites or high PD-L1 expression. This may help to identify beneficial populations for LCT. In this study, LCT improved mPFS by approximately 4 months and improved mOS by 6 months for patients with 1–2 residual lesions, but no significant benefit was observed for patients with more than 3 residual lesions. Therefore, LCT should not be widely recommended for patients with more than 3 residual sites after PD-1/PD-L1 inhibitor treatment, except for symptomatic relief.

A considerable body of literature has demonstrated the interaction between LCT and immunotherapy (14, 34). Radiotherapy (especially SBRT) is considered as being able to enhance the response to immunotherapy through several mechanisms, such as via the regulation of CD8+ T cells (34, 38). Residual disease after PD-1/PD-L1 inhibitor therapy may be resistant to PD-1/PD-L1 inhibitor therapy, and LCT of residual disease may release tumor antigens from dead cells, which may resensitize residual disease to PD-1/PD-L1 inhibitor by activating natural killer cells or CD8+ T cells. In our study, the survival benefit was mainly observed in patients with high PD-L1 expression. A possible explanation for this effect is that diseases with high PD-L1 expression are resensitized to immunotherapy by the strong immune stimulation resulting from LCT.

Many studies have demonstrated the feasibility and potential clinical value of local consolidative therapy combined with systemic therapy in treating metastatic NSCLC. Consolidative therapy to all metastatic sites has been shown to be a feasible option for patients with oligometastatic NSCLC during first-line targeted treatment, with significantly improved PFS and OS compared with LCT to partial sites or targeted treatment alone (39). In addition, another study showed that LCT to all lesions could significantly reduce the risk of disease progression and death compared with non-LCT, however, LCT to partial metastatic lesions failed to improve PFS and OS compared with non-LCT (40). To the best of our knowledge, most clinical trials have only included patients who received LCT for all metastatic lesions. Therefore, whether patients who receive LCT for only some lesions can exhibit improved survival time remains controversial, especially in the era of immunotherapy. Herein, our study demonstrates for the first time that there is no survival benefit for oligo-residual NSCLC patients who receive LCT to only some of the lesions after PD-1/PD-L1 inhibitors treatment.

This study had several limitations. First, this was a single-center study with a relatively small sample size and a retrospective design, which may have caused considerable selection bias in our study. Although the main clinical characteristics between the LCT group and the non-LCT group were generally balanced, some important baseline parameters were not included. In addition, it was difficult for us to collect accurate and comprehensive clinical information on the safety profiles of the two groups because they were not documented in detail. A larger prospective study with strict enrolment criteria is imperative to confirm these preliminary but valuable findings that were provided in our study and to further evaluate the safety and quality of life of LCT in PD-1/PD-L1 inhibitors-treated NSCLC patients with ORD.

In conclusion, LCT appears to provide additional survival benefits for NSCLC patients with ORD after effective PD-1/PD-L1 inhibitors-based therapy, especially for patients with 1–2 residual lesions, high PD-L1 expression or who receive LCT for all residual diseases. Prospective studies with more rigorous designs and larger sample sizes are needed for further validation.

## Data Availability

The original contributions presented in the study are included in the article/**Supplementary Material**. Further inquiries can be directed to the corresponding author.
