# Supplementary material for: The clinical value of local consolidative therapy for oligo-residual disease in PD-1/PD-L1 inhibitors-treated non-small cell lung cancer
Source: Front Immunol. 2024 Dec 17;15:1525236. doi: 10.3389/fimmu.2024.1525236 (PMC11685184; doi:10.3389/fimmu.2024.1525236)
Supplement: Supplementary file 1 [file Table1.docx]

Supplementary Material

**1 Supplementary Table1. Univariate analysis for PFS.**

|  | HR | 95% CI | *P* value |
| --- | --- | --- | --- |
| Age (≤55 vs. >55) | 0.869 | 0.564-1.339 | 0.524 |
| Gender (male vs. female) | 1.105 | 0.722-1.692 | 0.645 |
| Smoking (yes vs. no) | 1.166 | 0.757-1.796 | 0.487 |
| Pathology (non-squamous carcinoma vs.  squamous carcinoma) | 0.875 | 0.572-1.340 | 0.540 |
| Brain metastasis (yes vs. no) | 1.597 | 0.981-2.598 | 0.060 |
| Regimens (monotherapy vs.  combinational therapy) | 1.278 | 0.832-1.963 | 0.262 |
| ICIs (PD-L1 monoclonal antibody vs.  PD-1 monoclonal antibody) | 1.017 | 0.616-1.680 | 0.948 |
| Number of residual sites (1-2 vs. 3-4) | 1.183 | 0.760-1.842 | 0.457 |
| Number of metastatic organs (1-2 vs. >2) | 1.508 | 0.979-2.323 | 0.063 |
| PD-L1 level (high vs. low+unknown) | 1.278 | 0.817-1.999 | 0.283 |
| Local consolidative therapy (yes vs. no) | 0.591 | 0.373-0.937 | 0.025 |

**2 Supplementary Table2. Univariate analysis for OS.**

|  | HR | 95% CI | *P* value |
| --- | --- | --- | --- |
| Age (≤55 vs. >55) | 0.449 | 0.230-0.879 | 0.019 |
| Gender (male vs. female) | 0.969 | 0.520-1.806 | 0.921 |
| Smoking (yes vs. no) | 0.755 | 0.406-1.407 | 0.377 |
| Pathology (non-squamous carcinoma vs.  squamous carcinoma) | 0.965 | 0.520-1.793 | 0.911 |
| Brain metastasis (yes vs. no) | 1.169 | 0.582-2.349 | 0.66 |
| Regimens (monotherapy vs.  combinational therapy) | 1.141 | 0.608-2.141 | 0.682 |
| ICIs (PD-L1 monoclonal antibody vs.  PD-1 monoclonal antibody) | 1.148 | 0.560-2.354 | 0.706 |
| Number of residual sites (1-2 vs. 3-4) | 1.963 | 1.047-3.679 | 0.035 |
| Number of metastatic organs (1-2 vs. >2) | 2.032 | 1.061-3.894 | 0.033 |
| PD-L1 level (high vs. low+unknown) | 1.763 | 0.898-3.464 | 0.100 |
| Local consolidative therapy (yes vs. no) | 0.373 | 0.189-0.737 | 0.004 |

.
